# What is the patient re-identification risk from using de-identified clinical free text data for health research?

**DOI:** 10.1007/s43681-025-00681-0

**Published:** 2025-02-26

**Authors:** Elizabeth Ford, Simon Pillinger, Robert Stewart, Kerina Jones, Angus Roberts, Arlene Casey, Katie Goddard, Goran Nenadic

**Affiliations:** 1 Brighton and Sussex Medical School, Brighton, UK; 2 Akrivia Health, Oxford, UK; 3 King’s College London, London, UK; 4 South London and Maudsley NHS Foundation Trust, London, UK; 5 Swansea University, Swansea, UK; 6 University of Edinburgh, Edinburgh, UK; 7 University of Manchester, Manchester, UK

**Keywords:** Natural language processing, Clinical text, Health data, Data science, Privacy, Data governance; Confidentiality

## Abstract

Important clinical information is recorded in free text in patients’ records, notes, letters and reports in healthcare settings. This information is currently under-used for health research and innovation. Free text requires more processing for analysis than structured data, but processing natural language at scale has recently advanced, using large language models. However, data controllers are often concerned about patient privacy risks if clinical text is allowed to be used in research. Text can be de-identified, yet it is challenging to quantify the residual risk of patient re-identification. This paper presents a comprehensive review and discussion of elements for consideration when evaluating the risk of patient re-identification from free text. We consider (1) the reasons researchers want access to free text; (2) the accuracy of automated de-identification processes, identifying best practice; (3) methods previously used for re-identifying health data and their success; (4) additional protections put in place around health data, particularly focussing on the UK where “Five Safes” secure data environments are used; (5) risks of harm to patients from potential re-identification and (6) public views on free text being used for research. We present a model to conceptualise and evaluate risk of re-identification, accompanied by case studies of successful governance of free text for research in the UK. When de-identified and stored in secure data environments, the risk of patient re-identification from clinical free text is very low. More health research should be enabled by routinely storing and giving access to de-identified clinical text data.

## Introduction

Electronic health records (EHRs) have been used for at least three decades in large-scale research to create new knowledge to inform clinical care, practice and policy [1]. Clinical information is recorded into EHRs in different ways, with some entered in specific fields and formats within the EHR interface, and other information written in as free text or attached in documents such as outpatient letters. Structured clinical information is often captured in clinical coding systems such as SNOMED-CT or ICD-10, along with medication, referrals, tests and other labelled fields. Conversely, notes made at consultations, referral, outpatient and discharge letters, imaging or pathology reports and nursing notes are considered unstructured, as they are written in natural language and often without specific constraints in form or content [2].

In the course of everyday life, individuals constantly generate data through use of commercial services (e.g. store loyalty cards, mobile phone use) and through health services. In contrast to commercially generated data (such as purchasing patterns, phone call logs), health data is seen by most in society as particularly sensitive and confidential. For example in the European Union’s GDPR Article 9 [3], health, biometric and genetic data falls under a special category for processing regulations, requiring additional protection, and this is similar in other jurisdictions. Before using health data in research, the data usage needs to meet these regulations, particularly around de-identification and pseudonymisation to protect patient privacy. Different de-identification methods have been successfully deployed to support secondary use of structured health data. However, because of the challenges around de-identification for maximising patient privacy and data security, free text clinical information tends to be less available beyond the immediate clinical setting where it was produced, and may not be readily available for secondary uses such as research or service improvement [2]. However, clinical free text represents a vast, untapped source of rich information to guide research and clinical care, including patient-specific context and details that clarify and supplement information coded in structured data fields [4]. Furthermore, some clinical information valuable for research in mental health, pathology and imaging reports, is not available in coded structures but only in free text form. There are therefore ethical questions around not using text [5, 6], as restriction of EHR-based research to structured data may result two negative outcomes: firstly, impactful research being obstructed resulting in negative health consequences for patients, and secondly, unintended discrimination between clinical specialties resulting in wider health and social inequalities in the availability of clinically relevant evidence.

In this article, we consider the risks and safeguards around using free text clinical data for research, outside of the original clinical setting, particularly considering the United Kingdom (UK) legal context and infrastructure. We aim to conceptualise the risk of any patient being re-identified and the risk of harm to patients from potential re-identification. This provides a framework by which decision-making bodies might assess the risks posed by a data-access request for clinical text data. To give a context to this, we discuss the purposes for which researchers want access to free text and the regulations around this access. We then give a background to methods previously used for re-identifying health data and how successful these were. We also discuss the additional protections that are put in place around accessing health data, particular to the UK, and highlight recent work on public views of free text being used for research. Finally, we present real-world examples of two organisations that curate free text data. We use these to consider what good practice looks like in terms of de-identification, data security, and applying for data access to support researchers who endeavour to further medical knowledge by analysing this rich source of clinical information.

## Why do researchers want access to free text, what do they do with it, and how is it currently protected legally?

Free text data can address gaps of missing data in structured fields and increase the quality of research [7, 8]. It may also provide a closer representation of clinical reality than some sources of structured data such as codes applied to patients’ record for billing purposes [9]. Some healthcare domains use very little structured data in their patient records and reports, and researchers making use of routinely collected data in these health domains (e.g. mental health) must find ways of safely processing and analysing clinical text.

One of the benefits of using routinely collected health data is exploring issues at population scale. Some researchers want to read and manually code raw text for a qualitative thematic analysis. However, in our experience, this happens infrequently, as the volume of text is large, and manual analysis is not scalable. Instead, most researchers requesting access to clinical text want to structure information from the text for statistical analysis of trends in the population and relationships between variables. Therefore, most research applications to access and use free-text data have one of two aims:

1. To develop and train computer algorithms (natural language processing; NLP) to extract relevant clinical information from other volumes of text. This may be considered research in its own right or may be considered data infrastructure development or data processing. Commonly, this activity **does** involve researchers seeing a sample of clinical text. This is because—to train NLP algorithms—text must be marked up by annotators to indicate what are the clinically relevant words and phrases and to what concepts they belong. Sample volumes needed for training are decreasing, due to pre-trained large language models (LLMs), but often a reasonable quantity of text is still needed for the validation of models. Because of the historical difficulty of getting large corpora of clinical text on which to pre-train LLMs, available healthcare text algorithms are often in development phase, and researchers still need raw (de-identified) text to further fine-tune, check and validate accuracy for the specific task.
2. To run validated NLP algorithms on largely **unseen** text in order to extract information and structure it for statistical analysis to answer clinical research questions.

In many cases, requests for access to free text for humans to see, are for projects that are developing the clinical data processing infrastructure, rather than as clinical research in its own right. For actual clinical research, which will use the outputs of validated NLP models, often, little human contact with unprocessed data is needed [7], thus reducing the privacy risk. It is worth considering therefore if there should be different rules or approaches for these two activities, although both will need scrutiny from a data protection officer and to comply with the laws protecting data. While data processing (transforming raw data into a usable form) must happen in a lawful way, using data for health research (using processed data to draw conclusions, identify patterns, and make informed decisions) is usually subjected to additional review, such as by university or health board ethics committees, to ensure that the research will respect the dignity, rights, and welfare of any person affected by the research. In this article we focus on accessing the data with the purpose of NLP development to support future clinical research.

As stated above, in most jurisdictions, all health data is protected by laws such as the GDPR in the European Union, HIPAA in the United States, the Personal Information Protection and Electronic Documents Act (PIPEDA) in Canada and The Privacy Act 1988 in Australia. In England (as part of the United Kingdom (UK)), as well as the Data Protection Act 2018, English common law applies [10, 11], as well as other statutes such as the NHS Act 2006 and Control of Patient Information Act 2002 (which governs the use and disclosure of confidential patient information by healthcare professionals and organizations and provides a legal framework for the responsible use of patient information) [12]. In line with legal and ethical principle of data minimisation, the de-identification or anonymisation of health data is required before data can be used for secondary purposes beyond the provision of care, without individual patient consent. De-identification, legally synonymous in the UK and EU with pseudonymisation [13–15], describes the process of data transformation techniques including the removal of direct identifiers (name, address, date of birth, etc.) whilst retaining sequences of clinical events and processes with date stamps, which may be unique to the patient. To be considered anonymised under UK and EU law, data must generally be sufficiently processed or aggregated so that identifiability of the individual is unlikely, taking into account any means reasonably likely to be used. De-identified data remains within the scope of UK and EU law as personal data, whereas anonymised data falls outside the material scope of the law. Assessing whether data is de-identified or anonymised is complex, and data which is readily identifiable to one person, may be beyond the scope of identifiability to another [16–18].

## How is clinical free text de-identified and how accurate is this process?

By its nature, free text health data is highly likely to contain sensitive information and patients’ social context, including, for example, treatment choices and outcomes, family circumstances, confidential and sensitive personal information (e.g. a patient’s sex life or sexual orientation) etc. Letters and reports in particular routinely contain patient identifiers, both direct (e.g. name or address) and indirect (e.g. a unique combination of identifiers). As a consequence, clinical text must be suitably processed to de-identify data and protect individual privacy before the information can be shared with research teams who want to develop NLP algorithms. The challenge has been in developing effective methods^1^ to de-identify free text at scale whilst retaining data utility.

Most de-identification algorithms are assessed or validated by comparing their outputs to a human-marked-up gold standard test dataset which is not used in the training or development phase. The two common metrics are “recall” which is the proportion of relevant identifiers that were retrieved and redacted by the algorithm (equivalent to “sensitivity” in statistical parlance), and “precision” which is the proportion of true positives among the retrieved identifiers (equivalent to Positive Predictive Value (PPV) in statistical terms). The success of a de-identification algorithm might be measured by counting every single missed identifier (a “leak”) or by counting the number of documents which had one or more missed identifier. Different ways of conceptualising these metrics have been discussed in depth elsewhere [19].

We examine the development of clinical text de-identification starting in 2004, noting that other recent overviews are available [20, 21]. De-identification requires the algorithm to correctly recognise identifying information and either replace it with a generic placeholder (e.g. [X] or [patient name]) or a plausible but invented surrogate (e.g. a new, plausible name; an approach also known as Hidden in Plain Sight (HIPS) de-identification). Early de-identification methods for clinical free text relied largely on rule-based methods to redact or scrub identifiers, evaluating the methods against open-source databases such as MIMIC-II [22–24]. An early example was an algorithm called De-ID which was developed on MIMIC-II ICU nursing notes [25] and tested in multiple other data sources, including in Swedish [26] and French [27] hospital data, and Canadian Primary care data [28]. The best accuracy metrics recorded for this rule-based approach were recall = 0.97 and precision = 0.75; when ported to data in a new language, the performance dropped (recall up to 0.76 and precision up to 0.23). Some of the most successful subsequent rule-based methods have used regex (explanation of regex: [29]) and pattern-matching based on knowing the patient’s name for that particular document, drawn from a source EHR system. This method has been successfully used in the South London and Maudsley (SLaM) CRIS de-identification algorithm with precision and recall at 98% [30, 31], and in an Australian approach [32] which achieved a 100% de-identification rate for patient names.

Teams have also experimented with the Hidden in Plain Sight (HIPS) method [31, 33–37]. As mentioned, in this method the algorithm locates identifiers, and replaces them with credible surrogates (e.g. replacing a real patient name with a random name). Thus, the text still reads coherently as if identifiable information is in place. This approach has the benefit that if an identifier is not picked up by the system, the leak is hidden within the surrogates, and the reader cannot tell which of the identifiers might be leaked, which are the true values, and which are invented, reducing the re-identification risk.

Machine learning (ML) approaches to de-identification were developed from early 2000s, still using the scrubbing approach. These include systems such as MIST [38], BoB [39], and MeDS [40], which reached over 90% precision and recall in the held-out test data. A key event for de-identification was in 2014 when an Informatics for Integrating Biology & the Bedside (I2B2) challenge to develop de-identification algorithms on the MIMIC III dataset [41] was organised. This event produced a wave of new approaches, such as neural network machine learning, adversarial approaches, and ensemble learning. This challenge resulted in recall and precision scores in the range 0.97–0.99 [35–37, 42, 43]. A key barrier to further expanding and improving on machine learning methods is the need for new sets of training data in which real identifiers are marked up by a human annotator, so that the machine can learn from them. There is usually a block to this stage as researchers outside the patient’s direct clinical team are not allowed access to identifiable patient information, restricting who can mark-up text. Thus, even the newest English Language algorithms have often been developed on this single publicly available corpus—the 2014 MIMIC-III dataset [36, 37, 43–45]. However, de-identification algorithms have been developed in a range of languages and settings, including Dutch [46], German [47], Swedish [48], French [34, 49], and Chinese [33].

We have recently entered a new phase of natural language processing which is based on large pre-trained language models such as BERT [50] and GPT-4 [51]. These are trained on vast quantities of relevant language data using huge neural networks. They can be fine-tuned to perform specific tasks with only a few additional examples. Thus, studies showing they can be repurposed to perform de-identification tasks for clinical text are emerging [52, 53].

An alternative approach to obtaining real patient data for the NLP algorithm development stage is that of generating synthetic or semi-synthetic text data; these are datasets which are generated either from sampling and “mixing up” the original data or using statistical properties of an original dataset, to produce new patient records which do not belong to any real person. Synthesis methods have been developed to promote protection of privacy, allowing increased and faster access of researchers to healthcare research data, and to address the lack of realistic data for algorithm development and testing [54]. The simplest form of using synthesis is the previously referenced HIPS method where only identifiers are replaced with unreal pseudonyms. However, entire patient documents can be generated either by training a model on large back catalogues of patient records [55], or by feeding general large language models with appropriate prompts, and using generative AI to create fabricated documents [56, 57]. Synthetic data can then be used to train and validate natural language processing algorithms before sending the algorithms into real clinical settings. This means a human never needs to see true, identifiable clinical text before it is structured, thus bypassing the main privacy issues. Some teams are also working on generating synthetic text with identifiers, in order to train de-identification algorithms [58]. These approaches would work well as long as the properties of the synthetic data match and encompass all properties of the real data.

## What are the main routes to re-identification and how do risks differ for structured vs. unstructured data?

In this section we will consider how re-identification of individuals could be achieved by potential adversaries using clinical data and particularly free text. We define re-identification as a process of working out the person the data pertains to, rather than recognising a single identifier such as name or date of birth slipping through. There are three main routes to re-identification: (1) through disclosures or errors; (2) through unique sequences of events; (3) through breaking the pseudo encryption.

A first possible target for re-identification attacks are “leaks”, that is, disclosures or errors in de-identification (e.g. an identifier that has been missed in the de-identification process). Accredited researchers using free text data are usually required to report back to the dataset team when such a disclosure is identified in the data they are processing, so that leaks can be investigated and rectified. It is usually unlikely that the disclosure of one part of a name or a date of birth is sufficiently unique to enable re-identification of an individual. While use of a high quality de-identification standard, such as HIPAA’s safe harbour methodology [59, 60] to remove direct identifiers, may reduce the likelihood of such disclosures and subsequent identification, increasingly, critics argue that sole reliance on a de-identification standard to achieve patient privacy should be avoided, and additional protections are needed [61].

A second possibility for re-identification relies on the fact that, following removal of direct identifiers, there are likely to be unique sequences of events left in patient-level data represented by coded and/or free text clinical concepts with date stamps. One-off unique attributes may be revealing of particular patients (such as living on the 14th floor of a council run apartment block with a broken lift), as may rare events combined with dates (such as being released from prison on a particular date). These attributes are more likely to be contained in text data than in structured data. To use these unique features for re-identification, an intruder would need to know, or have access to, information about some other attributes of a particular individual, and be able to search for them in the original record or take an unusual set of patient information and search for other information sources that may be publicly available. This method would only work for re-identification of one individual at a time.

There is relatively little research-based evidence of how unique but indirect identifiers occur in text and how their occurrence is cumulative. DataLoch, one of the four regional Trusted Research Environments in Scotland, working as part of a collaboration, undertook a study to consider this question [62]. Using one year’s worth of discharge summaries from three major hospitals in Edinburgh, they applied NLP techniques to understand what type of contextual mentions have the potential to compromise a patient’s privacy. The team found prevalence and risks differed across age ranges. Some risks on their own would not reveal a patient’s identity (e.g. divorce, ages of children). However, as highlighted during the study’s public involvement workshops, public opinion was that some information (such as children’s ages) was too detailed and should only really be shared if it was relevant to the research. They also found that linked or multiple records from the same patient would increase the risk of identifiability.

For using combinations of features for re-identification at scale, researchers have hypothesized that the risk is measurable, but only when considering structured data, and particularly when it is possible to bring in and combine new datasets with the health data [63]. Firstly, an intruder would need to buy or acquire a dataset which had overlapping characteristics to the health dataset but also includes mappable identifiers (e.g. an electoral register). The risk of re-identification of one or more individuals increases with each variable or characteristic that overlaps between the protected dataset and the intruder’s identifiable dataset, and relies on individuals having unique combinations of characteristics; the more overlapping variables, the more likely there are to be unique combinations. The resulting risk is quantifiable with structured data containing variables in columns. It is however not quantifiable with unstructured text documents, because the number of health, medical or sociodemographic variables contained in the documents may vary from document to document, and are unknown until the documents are either read or automatically structured using computer processing.

The third method for re-identification is reversing the pseudonymisation or encryption key, e.g. working out how to reverse-engineer a hospital identity number from the unique pseudo-ID available in the dataset [64]. The risk for this method of re-identification would be the same whether structured or unstructured data is included in the dataset and depends on the strength of encryption for the pseudonyms, as well as the intruder perhaps having access to some examples of the correspondence between the pseudonyms and the true identifiers. All the above techniques are more likely to be successful if the attacker has more resources available to them.

A review of re-identification attacks on de-identified datasets found few attacks on health data have been attempted and publicised (six only; not involving clinical text); these were mainly carried out by researchers on public datasets to establish proof of concept. They found most re-identified data was not de-identified according to existing standards such as HIPAA [65]. Only one of the six re-identification attacks on health data was on a dataset that was de-identified according to one of the existing standards, and it was found that the risk of re-identification was very low (rate of re-identification was 0.00013) [66]. Other examples abound of supposedly anonymised or de-identified non-health datasets where individuals have been identified, such as Google search history or banking data [67].

While there are a number of ways to re-identify individuals when several datasets can be matched together [68], no additional re-identification methods have been described or explored in the academic literature when the ability to link in other data sources is carefully prevented and access is strictly controlled to legitimate researchers and analysts. As will be discussed below, hosting health data in a secure data environment can explicitly prevent the matching of another dataset containing identifiers and significantly reduce the likelihood of re-identification by providing a set of technical and organisation controls. Controls applied might include secured access, researcher vetting, encryption, policy controls, and controlled file egress and ingress.

## What else protects patients’ identities on top of de-identification?

Because even de-identified patient data may contain unique, and therefore potentially identifiable events or sequences, health data used for research is often protected by more mechanisms than de-identification alone. For example, some institutions in the US have created clinical data warehouses which securely store their patient record data, and, with an approved “umbrella protocol” (a waiver of authorization for HIPAA purposes), allow research on fully-identified clinical data [69]. Where there is no national consensus, interpretation of the same rules (e.g., HIPAA, GDPR) can differ by institution and result in substantially different practices.

In the UK since 2010, we have seen the diffusion of “Five Safes” (Safe Data, Projects, People, Settings and Outputs) principles from the Office for National Statistics (ONS) into the wider health research landscape resulting in a national consensus on how to store and use health data for secondary purposes [70]. “Five Safes” environments work like a *reference* library, where approved researchers have access to curated data in relation to their research via a strictly controlled environment, rather than a *lending* library where data might be sent out to researchers to host in their own organisation. These have taken form in Trusted Research Environments (TREs), now often called Secure Data Environments (SDEs) [71, 72] or Data Safe Havens. It is strongly recommended that all clinical text data made available for research is hosted in an SDE [2]. The authors know of no examples where de-identified UK clinical text has been made publicly available (e.g. been put on the internet), although this has happened in other countries (e.g. MTSamples [73] or MIMIC datasets).

An SDE ensures that data controllers retain control of the data: users are provided with only the data needed for the project that is approved, within a controlled computing environment, and data access can be revoked at any time. A system of accreditation is needed for researchers, who undergo information governance and “safe researcher” training [70] before being granted access; a data access agreement must be signed by the researcher’s employer and the researcher themselves. Because data is provided within the custodian’s controlled system, data use can be audited and managed, for example analytics and research outputs checked for true anonymity before being released, and the bringing in of any additional data sources can be prohibited [for more guidance and standards on how SDEs should function, see [74–76].

**Fig. 1:**
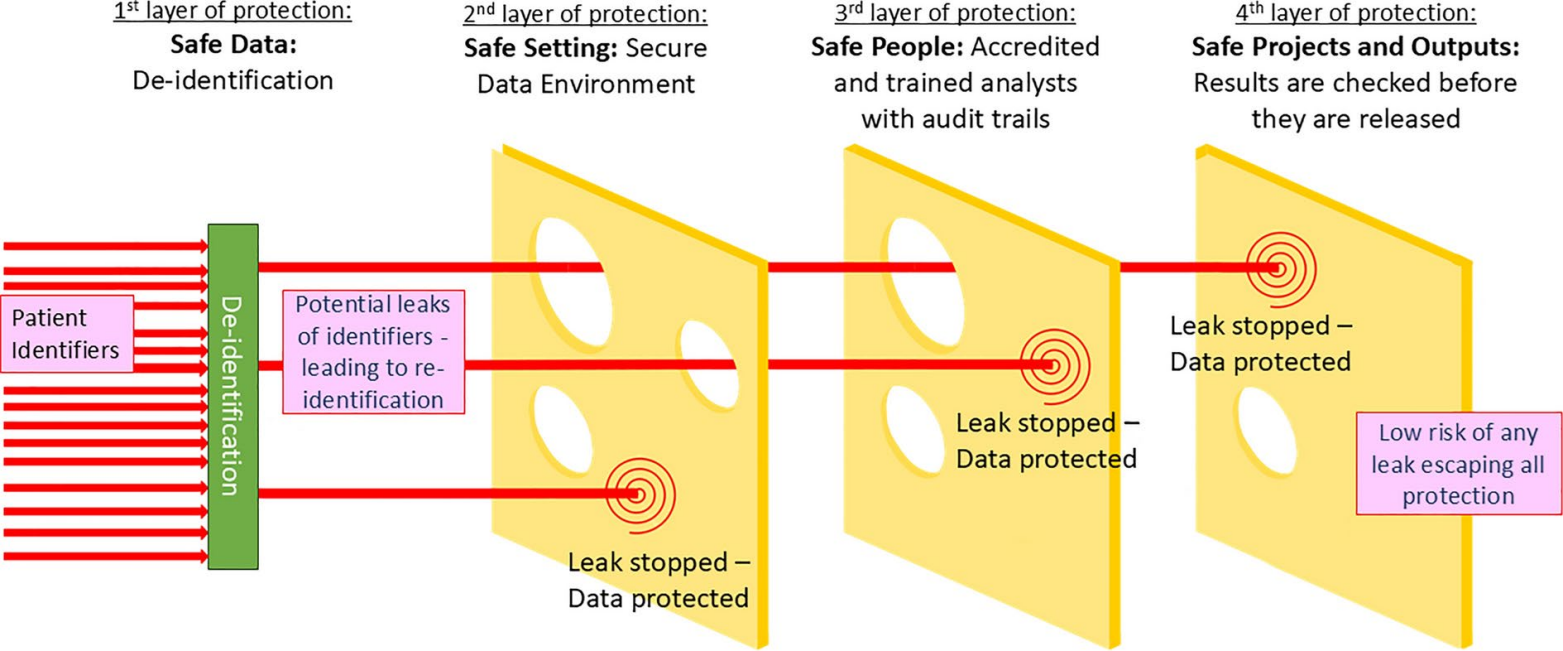
Swiss cheese model of security framework for clinical text data

It is therefore important to consider de-identification as only one layer in a series of protections that protect the privacy and confidentiality of individuals (see Fig. 1). A motivated intruder would need to pass all layers of protection to re-identify a patient from their clinical information held in text form. They would need to have a project approved, pass training and accreditation checks and be employed by a trusted organisation. Then, once they had access to the SDE, they would need to find identifiers or unique combinations particular to a person they would want to re-identify, and/or have an additional source of data to enable re-identification. SDEs are designed, technically, to prohibit the bringing or linking in of additional data sources without prior approvals, which would prevent any such re-identification happening at scale.

## Conceptualising risk of re-identification of de-identified text data in an SDE

Building on Scaiano et al. [19], we can formulate probability of re-identification (ReID) of a real person from Hidden in Plain Sight (HIPS) de-identified data held in an SDE is conditional on (i) the probability that any identifier is disclosed by the de-identification algorithm (a “leak”), multiplied by (ii) the probability that the attacker recognises a disclosure of an identifier, hiding in plain sight, given the leak, multiplied by (iii) the probability that a motivated attacker could gain access to the text data, given the recognisable leak.

We can put forward estimates for the base probabilities for each of these events: (i) The probability of a disclosure of any identifier is related to the inverse of the Recall metric of the algorithm used for de-identification, the best of which are around 0.97–0.99 [36, 37]. (ii) Two studies on HIPS de-identification have shown that identifying true leaks, even when clinicians look at their own re-synthesised data, is nearly impossible, so that the probability of recognising a true leak may also be close to zero [77, 78]. (iii) The design of the SDE system based on Five Safes principles means that the probability of a motivated attacker gaining access to the data is reduced to near zero, as researchers need to pass a number of screening points scrutinising their project, their training and assurances about their employer. It should be noted that the relationship between a disclosure, and the potential to re-identify from it, is not 1:1, making the above probabilities even smaller. Risk will vary by type of identifier and value (e.g. the mere exposure of a first name “James” would likely not result in an individual being identified in the UK because it is such a common name [79]). Thus, we get a sense that the probability of re-identifying an individual, given the best de-identification approaches and data being stored in an SDE, is extremely low and close to zero.

## How can we conceptualise harm from re-identification?

As a further consideration, while any re-identification from health records data requires robust action, the fact of being re-identified from health data does not necessarily cause harm to an individual. The potential routes to harm and types of harm therefore also need to be conceptualised and considered by decision makers in this space. In this section we consider possible harms at the individual, and collective or societal, level.

As summarised in Stockdale et al. [80], members of the public fear health data breaches occurring via hacking, data leakage or loss, or unauthorised access and use of data by the government [81]. The perception is that these data breaches could lead to harms to the individual including identity theft, unnecessary stigmatising judgements in clinical settings, consequences for employment, pension eligibility, or insurance costs, social discomfort and community embarrassment [80]. Lubarsky et al. [64] described that embarrassing information about an individual might be made public following re-identification. This could include medical history, sexual preferences, reproductive choices, details of conception or parentage; the disclosure of this kind of information to employer, spouse or community would cause distress to many individuals. The risks or harm which could come about from re-identifying people are likely to fall harder on people in minority groups, who are potentially more identifiable, and are vulnerable or disadvantaged in multiple ways [63].

While Article 82(1) of the GDPR provides a mechanism for individuals to bring claims against data controllers and processors where the individual has suffered material or non-material damages, a case has not yet been successful. In *UI v Österreichische Post AG*[82] the CJEU found that the “*mere infringement of the provisions of that regulation is not sufficient to confer a right to compensation.*” UK courts have reached similar conclusions in *Lloyd v Google* [83], suggesting that a tangible harm must be evidenced to win a data breach claim, and while such a harm may have occurred, so far no such case has been brought.

Harms can, however, also be considered from a societal point of view. For example, even if no particular individual is disadvantaged, some members of the public, when consulted, have felt that allowing parties with private or commercial interests to access public data would result in a collective disadvantage [84]. Some members of the public have expressed worry about the aggregation of data to discriminate against certain groups [81]. Others worry about the effect on confidentiality in healthcare; if patients feel their data is not secure, they might be less likely to disclose important clinical information, meaning that clinicians cannot make effective decisions about their care [85]. There could consequently be a societal loss of trust in the healthcare system as an organisation which has patients’ best interests at its heart. There have been highly publicised concerns over un-consented sharing of data with commercial partners, which have resulted in loss of trust in the NHS and government around data-sharing in the UK [86].

A US-based study which aimed to establish what harms had come to patients following re-identification from US-based health data, found that during 2019–2021, over 90 million health records were leaked or hacked (not specifically anonymised research databases, the majority were on internal healthcare servers or leaked by email) and reported to the Office of Civil Rights, but they could find no instances of patient re-identification or harm [6]. Similarly, there are no reports on patient harm or re-identification from the MIMIC III dataset, which has been de-identified and almost publicly available for many years. We also know of no cases of re-identification or harm coming to individual patients from the sharing of health data in the UK to date.

## What do we know about patients’ views of research using clinical free text?

A small number of studies and engagement activities have asked patients and the public how they feel about the use of their clinical free text data for research. A citizens’ jury asking informed members of the public about whether they would like clinical free text to be shared for research, concluded that overall they supported such an endeavour but made a number of recommendations for researchers and ethics committee members to follow [85]. The key suggestions for addressing outstanding concerns were:

1. Transparency: information must be made publicly accessible about how, when and under what conditions patients’ free text clinical data is processed and analysed, including giving patients access to published research in which their records were used.
2. Involvement: there should be a commitment to involve patients in elements of research and ethics decision-making.
3. Technology: the public wanted to see a commitment to continuous improvement in automated methods used for anonymising, coding and processing free text, and in IT systems safeguarding the data.

A second study discussed governance mechanisms for clinical free text with the public, and similarly highlighted a need for transparency and trustworthiness to build public confidence, with a joined-up approach to develop consistent standards, language and messaging [2]. It was also suggested that more information on the research benefits of using clinical free text is provided to the public (e.g. [7]). One patient representative described the current non-use of free text data as an “*atrocious waste*”; some highlighted the need for preserving the richness within health data by including free text, while others felt that patient involvement should be prioritised and be ongoing throughout projects [2].

A third study which included an exploration into how automation could be used in risk assessment and monitoring considered aspects of privacy assessment and minimisation of identifiable information in clinical free text. One of the key findings was that ‘public participants supported the idea of protecting patient privacy but raised a concern that removing or redacting from the data would negatively impact the quality and usefulness of the data [62].’

A further study asking participants about developing a donated databank of clinical text for NLP algorithm development, found overall broad support, but noted that patients *“expressed concerns around how inaccurate recording, the lack of up-to-date data, or subjective data based on a physician’s own impressions may threaten the aims of the databank, citing frequent instances of errors in their own health records”* [87]. The risk of being misrepresented in text data also concerned members of the citizens’ jury [85].

This suggests that patients who have been consulted are generally supportive of using health free-text data for both clinical research and NLP development, but with a strong commitment to public involvement and transparency in clinical text data projects being the main pillars on which public approval will rest.

## What does good practice around data de-identification, security and governance look like?

In this section we present two perspectives to show what good practice can look like. We first describe the Clinical Record Interactive Search (CRIS), a longstanding SDE, and give an overview of their platform working within the Five Safes framework and show how it has evolved, with patient involvement firmly embedded in the design. We also highlight the working model of Akrivia Health, a commercial entity, that provides health data curation and research services on behalf of NHS mental health trusts, to ensure safe de-identification, structuring and provisioning of mental health data for research.

### CRIS at South London and the Maudsley (SLaM)

The Clinical Record Interactive Search (CRIS) platform was developed in 2007–2008 in order to support researcher access to de-identified data, both structured and free text, from the EHR of the South London and Maudsley NHS Foundation Trust (SLaM), a provider of mental healthcare to a catchment of around 1.3m residents in four boroughs of south London [88, 89].

The CRIS security model [90] was developed by a patient-led group, and its continued operation (including review of all research applications) is overseen by a patient-led committee reporting to the SLaM Caldicott Guardian (the Caldicott Guardian is the senior person in an NHS organisation responsible for protecting the confidentiality of people’s health and care information). This security model has formed the basis for successive Research Ethics Committee approvals over the platform’s 15+ years of operation (current approval from Oxford Research Ethics Committee C, reference 23/SC/0257). It comprises not only the automated and regularly audited de-identification pipelines [30], but also processes for researcher and project approval, as well as data hosting (aligned to national ‘Five Safes’ principles) and patient and public dissemination (aligned to the National Data Guardian ‘no surprises’ principle). Over the years, the CRIS platform has developed an extensive programme of approved data linkages (with both health and non-health data) and a local SDE within the same firewall as the source EHR, to accommodate data hosting, processing and research use.

Clinical NLP development was adopted early in CRIS due to the limited information from structured fields alone in the mental health record. The CRIS security model includes specific protocols for clinical text access, both for manual coding or review, and for the development of NLP applications, all designed to minimise the risk of inadvertent re-identification. Use of text for NLP development requires a detailed template to be completed including the purpose and justification for the development, the quantity and nature of text required, access and processing environment specifications, NLP techniques and software, and data retention specifications. This approach facilitates both monitoring and routine auditing of text data use. Over 100 individual NLP applications have been developed over the past 10+ years, underpinning over 300 peer-reviewed publications to date, using CRIS data.

Patient involvement has been central to CRIS operation since its inception, not only through its original security model development and ongoing oversight, but also guiding research strategy and output. Building on a successful longstanding patient and carer advisory group trained to advise on CRIS data linkages and their use [91], and a time-limited group advising on CRIS priorities during the COVID-19 pandemic [92], a dedicated patient and carer group has been recently set up and trained over three sessions to provide guidance on CRIS NLP development and research use. These initiatives for mental health NLP consideration and guidance are also being adopted more widely by the Super Research Advisory Group assembled for the DATAMIND national hub for mental health data science [93].

### Akrivia Health Ltd

Akrivia Health is the spin-out company, from University of Oxford, of an NIHR grant-funded programme (CRIS-UK) which works with mental healthcare organisations (NHS providers) to de-identify and structure free text patient records using natural language processing, and facilitate research using Akrivia Health research ethics committee (REC) approved research database. At the current time 20 mental health trusts across England contribute data.

The Akrivia Health research platform is an example of a Five Safes environment which incorporates good governance and principles of data protection by design and default. All healthcare organisations using Akrivia Health’s platform remain data controllers for their data. They determine what data is extracted and can configure how data is de-identified at the point of extraction (alongside the application of local and national opt-outs). Akrivia Health is a data processor and has limited access to data for the purposes of maintenance and system delivery.

Akrivia Health provides a standard data protection impact assessment (DPIA) and governance model alongside their bespoke SDE, including a model local oversight committee (LOC), which each organisation can adapt to harmonise with any existing governance structures. The LOC, run by the healthcare organisation, is responsible for overseeing all research, reviewing research project applications, and ensuring that any other regulatory approvals are obtained where necessary. Once structured, data is available via the SDE with access controlled by health organisation system admins who can audit the use of the data.

Data is de-identified using a multi-layered approach. First, the algorithm masks free text relating to specific fields such as a first_name field that is then removed from free text data. This algorithm is supplemented with fuzzy logic and verification against nicknames. Additionally, bespoke regex coding is used to remove unusual strings of data. De-identified text data is used within Akrivia teams to generate NLP algorithms to extract relevant clinical information and therefore structure data. Usually only structured data is then presented back to the healthcare organisation in a secure platform, to enable statistical analysis for research and audit.

Public and patient consultation forms a key part of each healthcare organisation’s utilisation of the Akrivia Health platform. Akrivia Health work with each organisation to develop materials for informing the public about the organisation’s use of Akrivia, and processing of data. Organisations work with existing public engagement groups to understand their patients’ views about use of Akrivia services and adapt information materials correspondingly. Patient and public involvement (PPI) members are a frequent part of local oversight group membership to ensure a public and patient voice is included in the oversight of research. Akrivia also has its own PPI community with which it works to understand patient and public views to its proposed initiatives.

## Conclusions and recommendations

The UK healthcare text analytics community has been working for several years to address free-text data governance and privacy issues raised by data custodians, regulatory bodies and by patients and the public. In this paper we have considered the different purposes for which researchers want access to clinical free text; reviewed the accuracy of automated de-identification processes; discussed possible methods for re-identifying health data and the (lack of) success of these, and described the additional protections which are put in place around health data, particularly focussing on a United Kingdom perspective. We have described the theorised risks of harm to patients from potential re-identification and looked at research to determine whether the public support their clinical free text being used for research on an opt-out basis.

We have noted that clinical free text from patient notes and letters is most often processed and structured for large statistical sample analysis. Because of this, the need for researchers to access and actually read the full detail contained in clinical text is limited. Restricted text samples, however, are still needed to develop or refine computer algorithms for natural language processing, and will always be needed to assess the accuracy of these algorithms in new bodies of text in the future. However, as large language models come to dominate the NLP field, less and less raw text will be needed to develop accurate language processing models, which means that more text can be structured without researchers needing direct access, prior to use in research analysis. The less data that is available to researchers to view in full, arguably, the lower the re-identification risk.

We have shown the benefit to patient privacy of storing health data within secure data environments (SDEs), and strongly recommend that clinical text used for research is kept in these. When the layered protection of a Five Safes environment surrounds the clinical text, the protection offered by de-identification is important, but assessment of patient confidentiality and privacy should not rest on this alone. Through our conceptualisation of re-identification, we have shown that when we combine multiple protections, probabilities of an identifier slipping through each layer of protection is low, and the resulting possibility of a patient being re-identified from their clinical text becomes extremely low.

Currently missing from the health data sharing literature is any kind of balancing argument about the potential costs of preserving patient privacy. Maintaining the above described level of privacy for patients results in a range of expenses, both tangible (such as the cost of de-identification software and running secure data infrastructure) but also intangible, such as the delays and barriers to impactful research, resulting in discouragement of researchers and lost opportunity to improve healthcare and the health of the population [94]. While, as we have seen, the privacy risks are somewhat hypothetical, the costs of creating such highly secure datasets and environments are measurable. Some authors have argued that those who gate-keep access to medical research data are harming public health by imposing greater constraints on patient data than those required by the law, particularly where data is used for observational research and so activity is low-risk [5, 95, 96]. Some even go further and suggest that where patients have benefited from prior research in the form of new healthcare innovations, they have a moral obligation to share their data for research, if they are unlikely to be harmed by such data usage [97]. It would be interesting to see more balancing of these arguments in future work.

Over recent years we have generated insights into UK stakeholder views on free text data access and sharing [85], clarified the legal positions [2], and shown the clinical benefits of including clinical text in research [7], while UK national strategy has converged on a unified method for ensuring healthcare data privacy—the secure data environment [98]. We continue to find out more about public views, develop and improve technology for keeping data confidential and progress methods to ensure high quality extraction of relevant clinical information. As a promising way forward, we have proposed developing a national “donated” or consented databank on which technology for processing clinical free text can be developed and validated [87]. Using consented data circumvents some of the highlighted regulatory issues and would accelerate progress in text analytics infrastructure development.

It should also be noted that most endeavours within the healthcare text analytics community are working towards a time the involvement of researchers in the structuring of text data prior to statistical analysis is minimised. The aim is that, following NLP algorithm development, algorithms to structure text can be sent behind NHS computing firewalls with no human ever needing to see unconsented text data. Thus, some data protection officers (DPOs) consider the development of NLP algorithms as infrastructure development rather than research in and of itself. This activity is still subject to the same laws and scrutiny by DPOs as research, but in some use cases, does not need to undergo the full scrutiny of research ethics committees.

Public engagement activities have suggested that many members of the UK public are concerned that data is not linked up and utilised enough to improve services and manage healthcare resources efficiently [99]. Where data and privacy is sufficiently protected, the majority of people who have been consulted support the sharing of healthcare data for research for the public good [80]. With such a recent rapid increase in technology available for processing text at scale and in a standardised safe environment, now is the time for the UK and NHS to transition to regularly, routinely and safely using clinical information trapped in free text to improve healthcare for future generations.

## Data Availability

No datasets were generated or analysed during the current study.

## Footnotes

1 We note that de-identification methods are also NLP algorithms.
